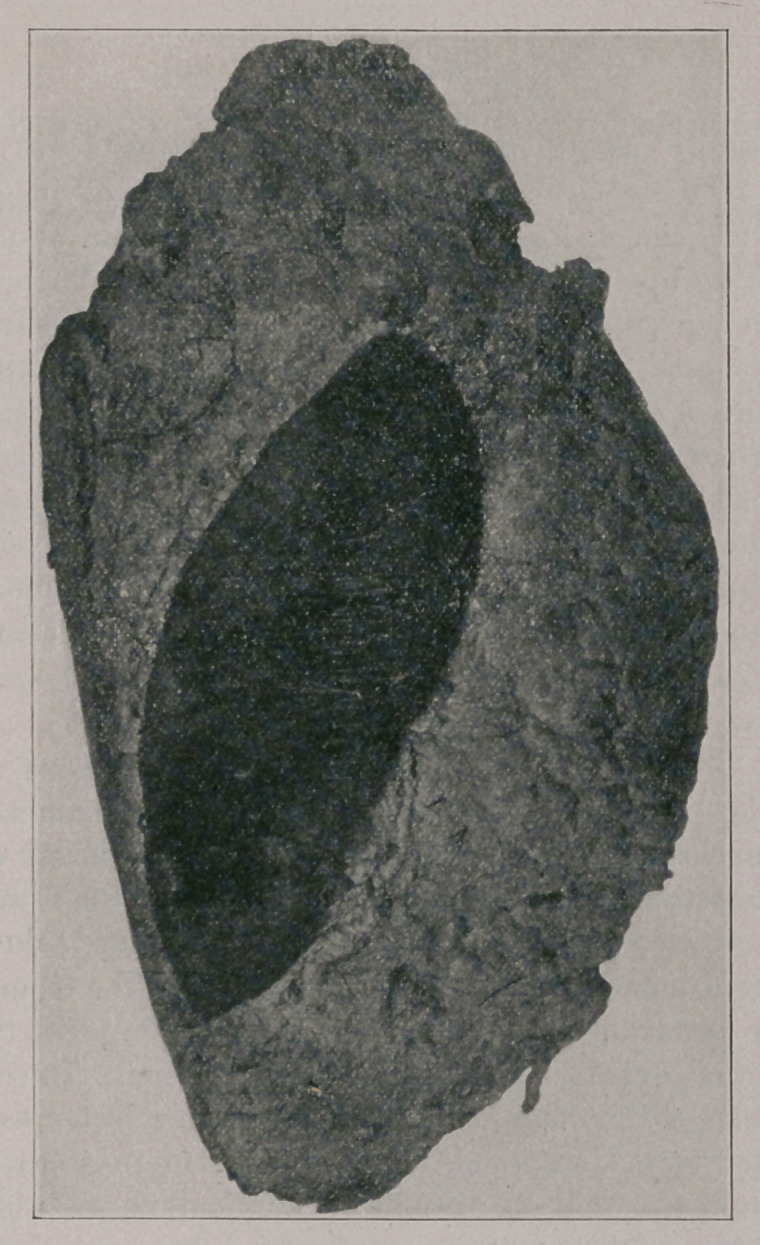# Lipoma of a Horse

**Published:** 1903-05

**Authors:** John J. Repp

**Affiliations:** Ames, Iowa


### LIPOMA OF A HORSE.

By John J. Repp, V.M.D.,
AMES, IOWA.

On March 30, 1900, a sorrel carriage gelding, eight years old, was brought to my clinic at the Veterinary Hospital, Iowa State College, to be treated for a swelling over the external face of the right anterior crural region which had been developing for a year. I found a smooth, flat tumor nearly a foot in diameter. It was not attached to the skin, but the skin showed some scars, which resulted either from ulceration or from attempts at treatment. The tumor was not prominent when the horse was standing, but, when he flexed the leg in advancing it became quite prominent when seen from the driver's seat, and on account of its unsightliness the owner wished to have it removed. The horse was cast on the grass and anæsthetized with chloroform. The hair was shaven over the tumor and the site washed with 5 per cent. carbolic acid. With a convex bistoury an elliptical incision was made to enclose a space eight inches long and four inches wide. The tumor was then dissected from the skin and the underlying tissues to which it was loosely attached, and removed. The tumor was circular in outline, about ten inches in diameter, three inches thick in the centre, and tapering to a very thin periphery. Its weight was four pounds eight ounces. It showed the characteristic lipomatous structure throughout, not being mixed with any other kind of tumor tissue. There was a well-developed reticulum of connective tissue enclosing lobules of adipose tissue one-eighth to one-half inch in diameter. It imparted an oily sensation to the hands, and the knife used in cutting it was smeared with grease. These latter features will always enable one to recognize a lipoma. The accompanying figure represents one-half of the tumor and shows the elliptical piece of skin which was removed with it. The photograph was made after the specimen had been kept in formaldehyde for several years.

On account of the large cavity resulting from removal of the tumor healing progressed slowly but uninterruptedly under daily washing with a disinfecting solution, until, finally, the wound closed up in such a manner as to leave only a small cicatrix to indicate that the locality had been the seat of a tumor.

---

Veterinarians in the East should be on their guard against one who goes under the name of Dr. A. Picquet, who claims to be a graduate of a French college, an ex-army veterinarian, and an acquaintance with many prominent veterinarians. He is an impostor, and not deserving of any assistance.

## Figures and Tables

**Figure f1:**